# Postoperative malrotation of humerus shaft fracture causes degeneration of rotator cuff and cartilage

**DOI:** 10.1038/s41598-021-98040-6

**Published:** 2021-09-20

**Authors:** Cheng Wang, Xiaoyuan Ma, Qiaohui Liu, Guofeng Dai

**Affiliations:** Orthopaedic Department, Qilu Hospital of Shandong University, No. 107 Wenhua West Road, Jinan, 250012 China

**Keywords:** Anatomy, Musculoskeletal system, Bone

## Abstract

We hypothesized that postoperative malrotation of humeral shaft fractures can alter the bio-mechanical environment of the shoulder; thus, rotator cuff and cartilage degeneration could be induced. Therefore, we designed an animal experiment to evaluate the impact of malrotation deformities after minimally invasive surgery for humeral fractures on the rotator cuff and cartilage, which has rarely been described in previous studies. Twenty-four New Zealand white rabbits were randomly divided into the sham control group (A), negative control group (B) and malrotated group (C). A sham operation with surgical exposure alone was performed in group A. Humeral shaft osteotomy was performed in Group B and C. In Group B, the fractures were fixed in situ with plate -screw system. While in Group C, iatrogenic rotational deformity was created after the proximal end of the fracture being internally rotated by 20 degrees and then subsequently fixed. The animals with bone healing were sacrificed for pathological and biochemical examination. In group C, the modified Mankin scale for cartilage pathology evaluation and the modified Movin scale for tendon both showed highest score among groups with statistical significance (P < 0.05); Disordered alignment and proportion of collagen I/III of rotator cuff were confirmed with picrosirius red staining; Transmission electron microscopy also showed ultrastructural tendon damage. Immunohistochemistry showed that both MMP-1 and MMP-13 expression were significantly higher in group C than groups A and B(P < 0.05). Minimally invasive techniques for humerus shaft fracture might be cosmetically advantageous, but the consequent postoperative malrotation could increase the risk of rotator cuff and cartilage degeneration. This conclusion is supported here by primary evidence from animal experiments.

## Introduction

Fractures of the humeral shaft account for approximately 1.31–3% of all fractures. Surgical intervention has tended to precede conservative treatment in recent years^1^. The surgical options for humeral shaft fractures include intramedullary nailing (IMN)^2^, open reduction and internal plate fixation (ORIF)^3^, minimally invasive plate osteosynthesis (MIPO)^4^ and external fixation^5^, which all have reliable outcomes. Owing to the advantages of minimally invasive characteristics, the MIPO technique and IMN have been widely accepted by both surgeons and patients. However, the postoperative deformity resulting from closed reduction using minimally invasive techniques has not been given adequate attention^6^, particularly for people who are active and have high-level requirements for sports, work or daily life.

Recently, the latent risk of malrotation deformity has been considered more important. Defects in muscle strength, changes in range of motion (ROM) and degenerative arthritis of the shoulder have been reported as consequences of postoperative malrotation of humeral shaft fractures^7^. Postoperative malrotation deformity might be inevitable since closed reduction is performed with guidance from only intraoperative fluoroscopy. As previously reported, postoperative malrotation of over 20 degrees was found in 27.2% in the IMN group (all patients had humeral head internal rotation) and 40.9% in the MIPO group (55.6% of patients had internal rotation and 44.4% had external rotation)^8^. Flury et al. reported that degenerative shoulder arthritis was present at 14.5 years of follow-up in 91% of patients who underwent a Weber internal rotation osteotomy^9^. In particular, serious shoulder arthritis of grades 3 or 4 developed in those with internal rotation exceeding 20 degrees. This finding was explained by impingement between the malrotated humeral head and the glenoid edge as well as the increased articular contact stress; nevertheless, the impact of malrotation on soft tissue around the shoulder (especially the rotator cuff) has not received adequate attention in previous studies.

Rotator cuff tears (RCTs) of the shoulder are thought to be the most common non-trauma cause of upper limb dysfunction in people over 50 years of age^10^. While the mechanism and pathogenesis of RCT remain unclear, genetic predisposition, extrinsic impingement and biomechanical imbalance from surrounding structures and intrinsic degeneration within the tendon are considered the most likely relevant factors. After a review of more than 700 studies, Zaid MB concluded that scapular anatomy parameters, as measured by the acromial index (AI), critical shoulder angle (CSA), lateral acromial angle (LAA), and glenoid inclination (GI), appeared to be significantly associated with rotator cuff tears as well as glenohumeral osteoarthritis^11^; this suggests that RCTs could probably be induced by anatomic variance.

We hypothesized that malrotation deformities could alter the anatomic parameters of the shoulder, such as the interspace between the tuberosity and the acromion, the coracoid process, and the coracoacromial ligament and the head centration on the glenoid. Thus, the force balance of surrounding muscles and the bio-mechanical environment of the shoulder could be disrupted, and theoretically, the deformity could lead to simultaneous soft tissue and bone degeneration. Therefore, this study aimed to evaluate the impact of proximal humerus malrotation on glenohumeral articular cartilage and the rotator cuff in a rabbit model. The study was carried out in compliance with the ARRIVE guidelines.

## Materials and methods

### Ethics statement

All experiments followed The “Principles of Laboratory Animal Care” (National Institutes of Health publication no. 80–23, revised in 1996) and the “EC Directive 86/609/EEC”. This study was approved by Laboratory Animal Ethics Committee of Hangzhou HIBIO Technolgy Co.LTD. entrusted by Qilu Hospital of Shandong University(Approval No.HB1705014). All efforts were made in accordance with the 3R principle (refine, reduce, and replace) regarding the care and use of laboratory animals. Thus, the experiment was carried out with n = 8 animals per group.

### Modelling of Postoperative malrotation

**Figure 1:**
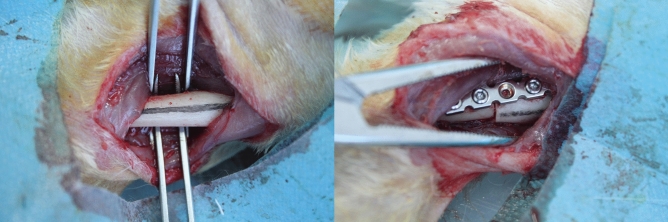
Intraoperative exposure of the humerus shaft and internal fixation after osteotomy.

**Figure 2:**
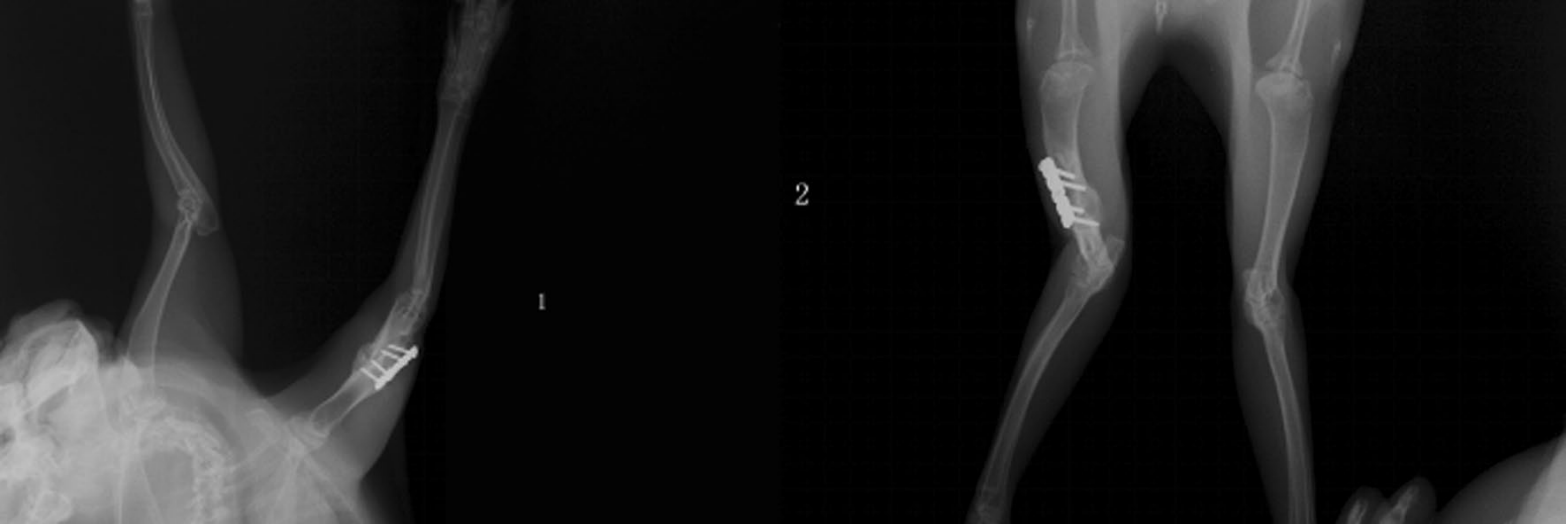
Postoperative X-ray analysis to confirm bone healing.

**Figure 3:**
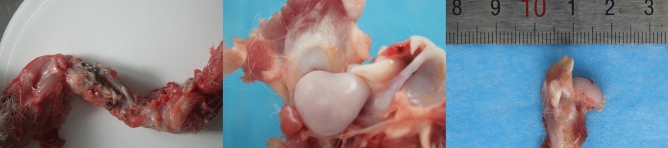
Specimens of the shoulder complex and proximal humerus with subscapularis tendon.

Twenty-four New Zealand healthy white rabbits (Aged 5–6 months, weighing 2.5–3 kg and a 50:50 ratio of males to females; Purchased from Hangzhou Yuhang Kelian rabbit Co., Ltd., license No.: SCXK (Zhejiang) 2013–0055, Certificate No.: 1706160010/1710100012) were randomly divided into the sham control group (A), negative control group (B) and malrotated group (C). Osteotomy of the humerus shaft and internal fixation were performed in group B and group C, while a sham operation was performed for the blank control group (A). The rabbits were fixed on a customized frame, and anaesthesia was given though the ear edge vein with 3% sodium (1 ml/kg). A 4- to 6-cm anterior-middle longitudinal incision was made along the humerus. The muscle was bluntly separated along the muscle fibre until the humerus was exposed to prevent injury to blood vessels and nerves. After marking the long axis of the bone surface with methylene blue, horizontal osteotomy was performed at the mid-shaft level. In group B, the fracture was fixed in situ. In group C,two paralleled Kirschner wires were fixed in the distal and proximal ends of the osteotomy plane respectively, and then the proximal Kirschner wire was internally rotated along the plane perpendicular to humeral shaft axis until the target angle (20 degrees) between the two wires was attained. Then the fracture was fixed with plate-screw system (Fig. 1). In group A, the animals underwent only surgical exposure without osteotomy and internal fixation. The animals were immediately immobilized by plaster casting, kept warm until they woke up from anaesthesia, and were then put back into separate cages and fed. Penicillin was intramuscularly injected at 1.6 million IU daily for three consecutive days. The plaster was removed after 6 weeks, and then weight-bearing activities were allowed. The animals were encouraged by robots to passively move in a small room for 1 h per day. X-ray screening was performed to confirm bone healing at 8 weeks after surgery (Fig. 2). At 20 weeks after surgery, the animals were sacrificed, and the shoulder with the rotator cuff was collected for pathological and biochemical examination (Fig. 3). All 24 animals had a postoperative stress response of a poor diet and reduced activity and recovered to normal 1 week later. In the process of modelling, 1 rabbit died from diarrhoea, and the procedure failed in 3 rabbits due to nonunion; therefore, 20 animals (8 from group A, 6 from group B and 6 from group C) were finally sampled.

### Histologic evaluation

Subscapularis tendon-bone complex and humeral head specimens were fixed in 4% formaldehyde solution for 3–5 days, decalcified in 10% EDTA solution for 1 month, fixed in 4% formaldehyde solution for 3–5 days, trimmed to an appropriate size, dehydrated with ethanol and embedded in paraffin. After staining with haematoxylin and eosin (HE;Sigma; Batch number: SLBN3249V for Haematoxylin and 62R80915X for Eosin), safranin O (Solarbio; Batch number: 20190926), or picrosirius red (Sigma; Batch number: #MKBK7017V). 4 µm sections were observed with light microscopy (BX43; OLYMPUS) and polarized light microscopy (EclipseCi-POL; NIKON).

The cell morphology and fibre arrangement of the tendons were evaluated under light microscopy with HE staining and polarized light microscopy with picrosirius red staining^12–15^. Transmission electron microscopy was used to observe the ultrastructure of cells. Two semiquantitative scoring criteria were respectively used to evaluate the pathological changes of cartilage(modified Mankin scale) and tendon (modified Movin scale)^16,17^.

### Biochemical analysis

#### Real-time quantitative PCR

Total RNA was extracted from tissues by a TRIzol centrifugal column method. After purity determination (microspectrophotometer; Merinton USA; SMA4000) and quantification, reverse transcription and quantitative PCR amplification (PCR Instrument, BIO-RAD USA; CFX Connect Real-Time System) were performed. The primer sequences are listed in Table 1. The mRNA expression of matrix metalloproteinase 1 (MMP-1), MMP-13 and stromal-derived factor 1α (SDF-1α) in articular cartilage was analyzed.

**Table 1:** PCR Primer sequences.

Gene	Sequence (5′–3′)
SDF-la-rabbit-forward	GATGCCCCTGCCGATTCTT
SDF-1a-rabbit-reverse	GTTGCTCTTCAGCCGTGCC
MMP13-rabbit-forward	GCCATTCCTTAGGGCTTGA
MMP13-rabbit-reverse	GGTGTTTAGGGTTGGGGTCT
MMP1-rabbit-forward	TGATGTGGCTCAGTTCGTCC
MMP1-rabbit-reverse	TGGTGAATGTCAGGGGTGT
Rabbit-GAPDH-forward	TGCCGCCTGGAGAAAGC
Rabbit-GAPDH-reverse	CGACCTGGTCCTCGGTGTAG

#### Western Blot technique

Total protein was extracted from tissues, and the protein concentration was determined by the BCA method (SPECTRA max Plus 384, Molecular Devices USA). After poly-acrylamide gel electrophoresis (Mini-Proten Tetra System, Bio-Rad USA) and incubation with antibodies, the chemical photosensitive mode of a gel imaging analyser was used (ChemiDoc XRS + System, Bio-Rad USA). The expression levels of MMP-1, MMP-13 and SDF-1α in articular cartilage samples were measured.

#### Immunohistochemistry technique

The sections were dewaxed with xylene and rehydrated. After antigen retrieval and incubation with antibodies (primary antibody: Abcam; secondary antibody: Beijing Zhongshan Jinqiao Biotechnology Co., Ltd), DAB and haematoxylin staining were performed. Fluorescence was observed under a microscope (BX43; OLYMPUS). MMP-1 and MMP-13 expression in cartilage was evaluated.

### Statistical analysis

Statistical analysis was conducted using SPSS 23.0 software. The Mann–Whitney test was applied for abnormally distributed data. Continuous data are described as the mean ± standard deviation (SD). Variables between/among the groups were compared using the Mann–Whitney-Wilcoxon test and one-way ANOVA. P-values less than 0.05 were considered significant, and P-values less than 0.01 were considered highly significant.

## Results

### Histological characterization

HE and safranin O staining results: No pathological abnormalities were found in the sham control group (A). In the negative control group (B), necrotic bone tissue and inflammatory cell infiltration were observed, and tendon fibres were arranged normally without pathological changes. In the malrotated group (C), inflammatory cells, which mainly included neutrophils and eosinophils, were more frequently observed than in other groups. Clearly proliferating tendon fibroblasts were observed among disordered tendon fibres. Group C lacked a superficial layer of cartilage and exhibited sparsely arranged chondrocytes, weakened matrix staining and disrupted tidemarks.

The slides were assessed by two authors who were both blinded with group assignment. Two semiquantitative scoring criteria were respectively used to evaluate the pathological changes of cartilage and tendon. 4 parameters of modified Mankin scale for cartilage included: cartilage structure, cellularity, proteoglycan depletion and tidemark integrity. The total score ranged between 0 (intact) to 14 (severely destroyed) (Table 2). And 7 variables of modified Movin scale for tendon included: fiber structure,fiber arrangement, rounding of the nuclei, regional variations in cellularity, increased vascularity, decreased collagen stainability, and hyalinization. And a 4-point scoring system was used, in which 0 indicates a normal appearance, 1 indicates a slightly abnormal appearance, 2 a moderately abnormal appearance, and 3 markedly abnormal appearance. The total score ranged between 0 (normal tendon) to 21 (most severe degeneration) (Table 3).

**Table 2:** Modified Mankin Score for cartilage.

**Structure**	
Normal	0
Irregular surface, including fissures into the radial layer	1
Pannus	2
Superficial cartilage layers absent	3
Slight disorganization (cellular rows absent, some small superficial clusters)	4
Fissures into calcified cartilage layer	5
Disorganization(chaotic structure, clusters,osteoclast activity)	6
**Cellular abnormalities**	
Normal	0
Hypercellularity, including small superficial clusters	1
Clusters	2
Hypocellularity	3
**Matrix Staining**	
Normal/slight reduction	0
Staining reduced in radial layer	1
Reduced in interterritorial matrix	2
Only present in pencellular matrix	3
Absent	4
**Tidemark**	
Intact	0
Destroyed	1

**Table 3:** Modified Movin Score for tendon.

Items	Score(points)
Fiber structure	Normal(0)
	Slightly abnormal(1)
	Abnormal(2)
	Markedly abnormal(3)
Fiber distribution	Normal(0)
	Slightly abnormal(1)
	Abnormal(2)
	Markedly abnormal(3)
Rounding of the nuclei	Normal(0)
	Slightly abnormal(1)
	Abnormal(2)
	Markedly abnormal(3)
Regional variations of cellularity	Normal(0)
	Slightly abnormal(1)
	Abnormal(2)
	Markedly abnormal(3)
Vascularity	Normal(0)
	Slightly abnormal(1)
	Abnormal(2)
	Markedly abnormal(3)
Decreased collagen of stainability	Normal(0)
	Slightly abnormal(1)
	Abnormal(2)
	Markedly abnormal(3)
Hyalinization	Normal(0)
	Slightly abnormal(1)
	Abnormal(2)
	Markedly abnormal(3)

**Figure 4:**
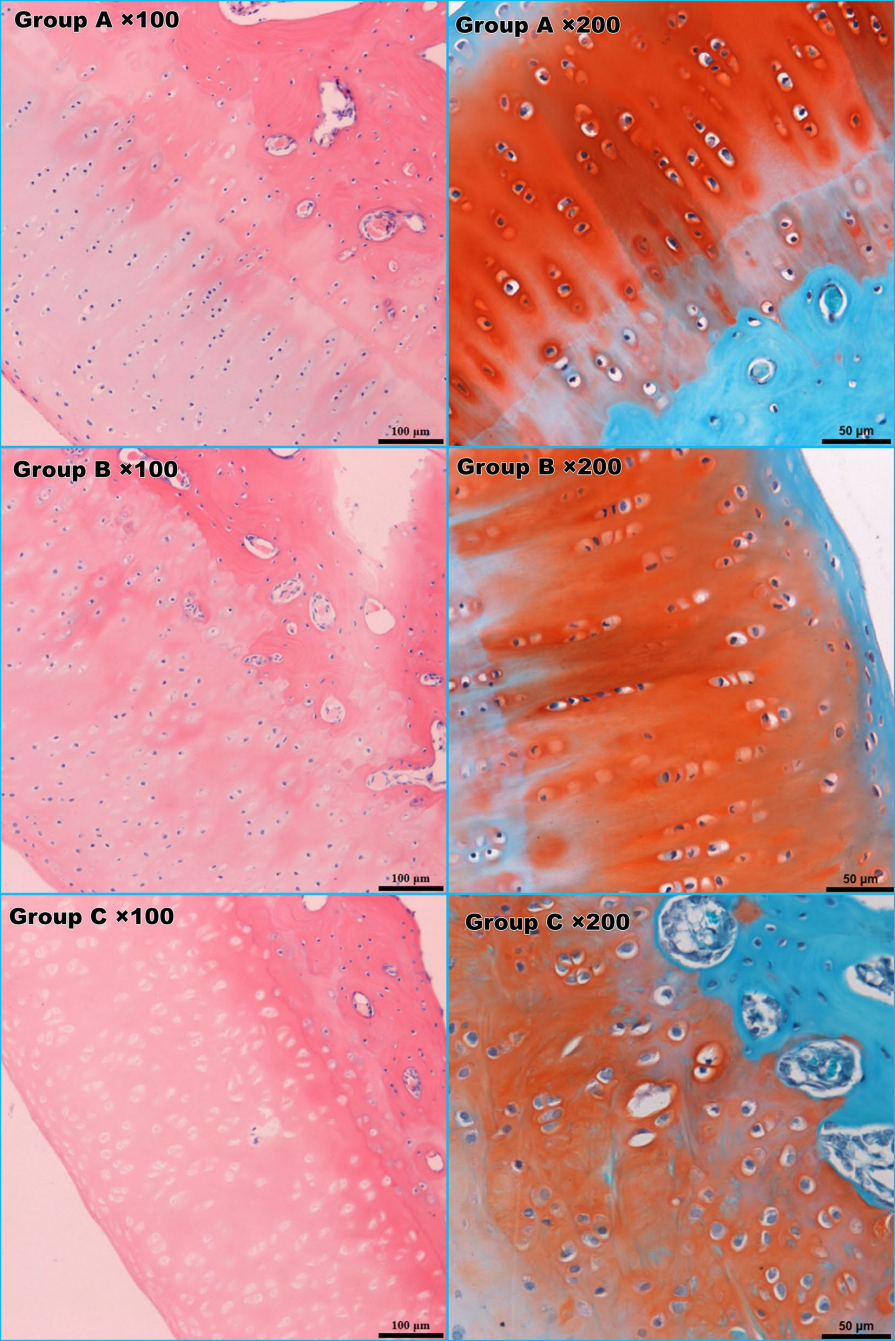
HE and safranin O staining of the cartilage. Group C lacked a superficial layer of cartilage and exhibited sparsely arranged chondrocytes, weakened matrix staining and destroyed tidemarks.

**Figure 5:**
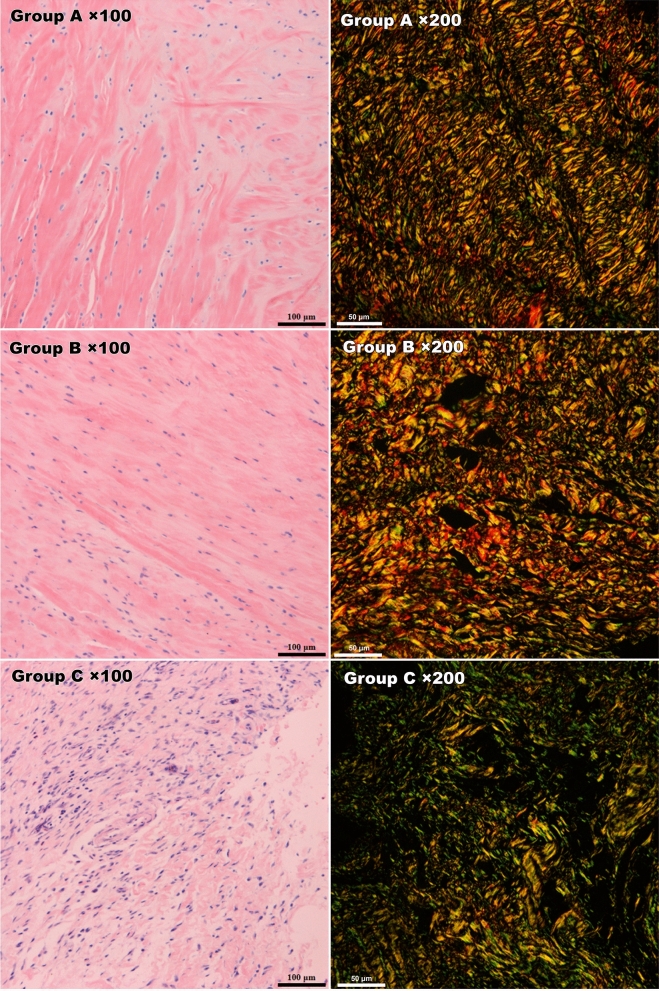
HE staining and picrosirius red staining of rotator cuff tissues under microscopy. Fibroblast proliferation and inflammatory manifestations were observed in group C (left column). Under polarized light microscopy, the overall alignment and proportion of collagen I/III in group C obviously differed from those in groups A and B based on Sirius red staining (right column).

The modified Mankin score of cartilage and modified Movin score of tendon both were significantly different between groups B and C (Mankin score:0.67 ± 0.52 Vs 2.14 ± 1.35 and Movin score: 2.25 ± 1.0 Vs 11.2 ± 7.3; P < 0.05) (Fig. 4, 5).

Picrosirius red staining of the tendon tissue was evaluated under a polarized light microscope. The collagen I fibres were thick, exhibited strong double refraction, and were a red or orange colour, while the collagen III fibres were thin, had weak double refraction, and were a green colour. The proportions of collagen I and collagen III among the groups were significantly different (A: 59.83 ± 8.37; B: 6.16 ± 1.63; C: 1.99 ± 1.37). The experimental group (C) presented a significantly higher proportion of collagen III than groups A and B (P < 0.01) (Fig. 5).

**Figure 6:**
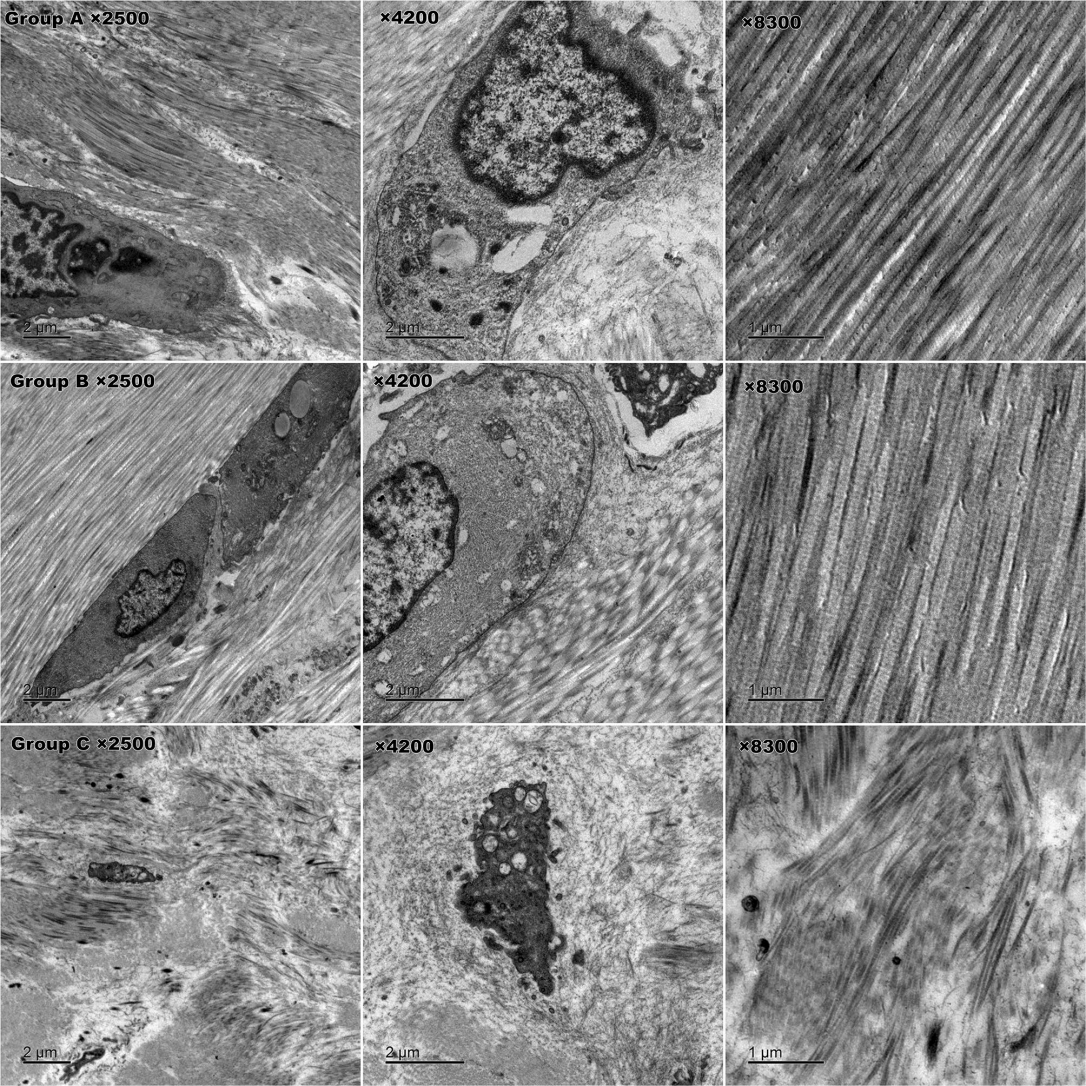
Ultrastructure of rotator cuff tissue under transmission electron microscopy. In group C, nuclear chromatin pyknosis with indistinct cytosolic organelles and distorted or broken fibrils were observed, which significantly differed from groups A and B (bottom line).

The ultrastructural changes in the rotator cuff were observed by transmission electron microscopy. In group B, tendon cells were slightly pyknotic with an increased number of pinocytic vesicles in the cytoplasm. Some cells demonstrated distention of the rough endoplasmic reticulum and lipid droplets. Fibrils were arranged in the intercellular space in a slightly loose manner. In group C, obvious nuclear chromatin pyknosis with indistinct cytosolic organelles was observed, and many pinocytic vesicles remained. The fibril arrangement was disordered and even broken (Fig. 6).

### Biochemical analysis

#### Quantitative real-time PCR

The mRNA levels of MMP-1, MMP-13 and SDF-1α in the cartilage were detected by qPCR and analysed with BIO-RAD CFX Manager 3.1 software. Glyceraldehyde-3-phosphate dehydrogenase (GAPDH) was used as an internal control. Compared with group A, groups B and C had increased levels of MMP-1 mRNA expression (no significant difference, P > 0.05) and decreased levels of MMP-13 mRNA expression (significant difference, P < 0.05). No difference in MMP-1 and MMP-13 levels was found between groups B and C. Moreover, the SDF-1α mRNA levels tended to be lower in group C than groups B and C (0.05 < P < 0.1).

#### Western blot technique

**Figure 7:**
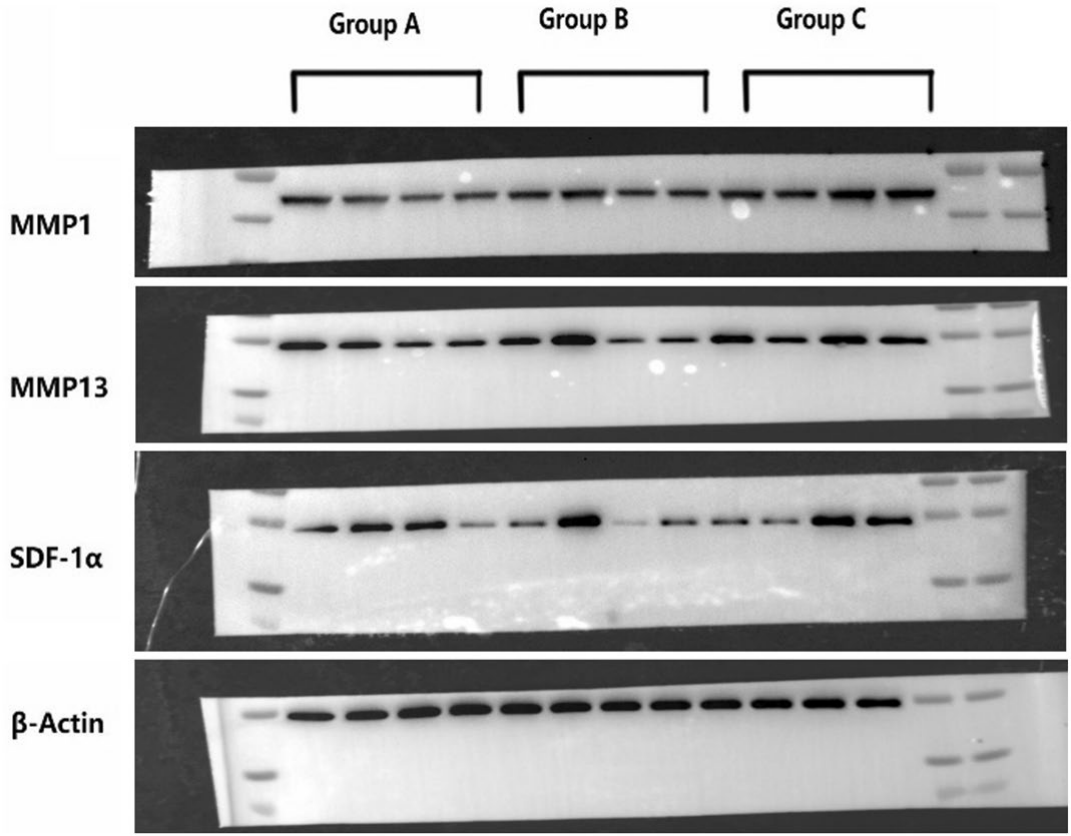
Western blot results(4 samples from each group) . Group C demonstrated the highest levels of MMP1, MMP13 and SDF-1α protein expression among the groups. Full-length blots/gels are presented in Supplementary Fig. 7.

Four cartilage samples were randomly selected from each group for Western blot evaluation. β-actin was used as an internal control, and group C demonstrated the highest levels of MMP1, MMP13 and SDF-1α protein expression among the groups; however, the results were not significant (Fig. 7).

#### Immunohistochemistry technique

**Figure 8:**
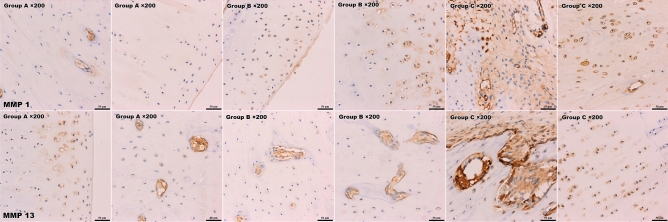
IHC of MMP-1/13. Both MMP-1 and MMP-13 were expressed at significantly higher levels in group C than groups A and B. MMP-1- and MMP-13-positive cells are shown in brown.

An immunohistochemistry (IHC) quantitative scoring system was applied to evaluate the IHC results. Five different visual fields (× 200) were randomly selected from each section for evaluation. The staining intensity was characterized as follows: 0 for no obvious staining, 1 for slight staining, 2 for moderate staining and 3 for strong staining. The percentage of positive cells was calculated as follows: 0 for up to and including 5%, 1 for 6–25%, 2 for 26–50%, 3 for 51–75%, and 4 for > 75%. The final score was calculated as the sum of the staining intensity and the percentage of positive cells. Immunostained MMP-1- and MMP-13-positive cells appear as brown under a microscope. Both MMP-1 and MMP-13 were expressed at significantly higher levels in group C (5.89 ± 0.6) than group A (3.0 ± 0.71) and group B (3.56 ± 0.53). Additionally, the staining score in group B tended to be higher than that in group C (0.05 < P < 0.1) (Fig. 8).

### Power analysis

We have determined the sample size based on literature of the histologic evaluation of rotator cuff and tendon researches on rabbit model. As listed in references^18–20^ the sample sizes of each group adopted ranged from 5–10. Also with PASS 2011 software (NCSS, Kaysville, UT), we found the sample size of 6 could achieve an over 90% statistical power. In our research, we performed power post-hoc analysis and the value is 84.3%.

## Discussion

Due to the surgical requirements, we attempted to use larger animals in this study. Previously, many animal shoulder models have been reported in the literature that involve rats, rabbits, goats, and sheep^21^. The rat model was the most extensively studied model because the anatomic relationship of the supraspinatus to the acromion in the rat shoulder is analogous to that in humans and it is cost effective. However, limitations and deficiencies of rat models have gradually been recognized in recent years. The shoulder of quadruped rats is unlike the human non-weight-bearing joint, and the small size of the rat shoulder increases the operational difficulty for the bone and surrounding tissues^22^. Furthermore, the portion of the rat supraspinatus that passes under the acromial arch is muscular but not tendinous like in humans^22^. Robert C Grumet studied a rabbit model to confirm that the relationship of the rabbit subscapularis tendon and scapular bony tunnel is analogous to that of the human supraspinatus, which passes under the bony acromion prior to its insertion on the greater tubercle of the humerus^21^. The author concluded that the anatomic architecture, histological properties and mechanical characteristics of the rabbit subscapularis muscle are similar to those found in the human rotator cuff^21^. Thus, rabbits were considered a suitable animal model to research rotator cuff pathology, especially that caused by extrinsic factor alterations (e.g., biomechanical changes)^22^. In recent years, rabbit models have been extensively applied in rotator cuff repair research^23–27^.

Severe osteoarthritis of the shoulder and irreparable RCTs are extremely challenging to treat in clinical practice. Presently, the results of total shoulder replacement are far from satisfactory^28,29^. Therefore, increasing attention has been paid to the mechanism of shoulder joint degeneration in recent years. Anatomic factors associated with shoulder glenohumeral osteoarthritis have been identified. Moor BK suggested that individual quantitative anatomy may imply biomechanics that are likely to induce specific types of degenerative joint disorders^30^. Meyer DC found more horizontal acromial orientation in the sagittal plane and increased posterior glenoid version in osteoarthritis of the shoulder were associated with eccentric, posterior glenoid wear^31^. Regarding iatrogenic anatomical alterations, Flury MP reported that 91% of patients who underwent internal osteotomy of the humerus suffered severe shoulder osteoarthritis in the long term^9^. A significant relationship was found between increased internal rotation of the humeral head and disease severity. Anatomical differences were also found to be correlated with RCTs. Zaid MB reported that an increased CSA and AI were correlated with RCTs, whereas a lower CSA appeared to be related to the presence of glenohumeral osteoarthritis^11^. Miswan MF reported that the acromioglenoid angle (AGA) was an excellent predictive parameter for diagnosing RCTs and glenohumeral osteoarthritis^32^. Moor BK reported that the acromion index, LAA, and CSA accurately predicted the presence of degenerative RCTs^33^. At present, few studies concerning the relationship between iatrogenic anatomical alterations and rotator cuff degeneration are available. Therefore, we designed this trial to highlight the potential impact of postoperative malrotation of humerus shaft fractures on rotator cuff pathology.

Histological evaluation showed a higher modified Mankin score and Movin score in group C. We believe that the altered anatomy could have caused joint instability, internal impingement or abnormal contact stress distribution, which may lead to cartilage damage. More noticeably, changes in rotator cuff pathology were observed. Group C showed inflammatory manifestations and obviously disordered collagen alignment, which was prominently shown with picrosirius red staining and transmission electron microscopy. These findings indicate that the inflammation caused by biomechanical imbalance and abnormal abrasion or impingement could disrupt the microstructure of the rotator cuff tendon and gradually develop into tendonitis or rupture. These findings need to be given more attention. Since proteins play a principal role and serve as the end effectors of cell functions, Western blotting and IHC provide more meaningful results than qPCR. Western blot analysis showed that MMP1, MMP13 and SDF-1α protein expression were higher in group C than the other groups, and IHC showed that both MMP-1 and MMP-13 were expressed at significantly higher levels in group C than groups A and B. These results indicated that cartilage degeneration occurred and were in accordance with the histological findings.

The limitations of this study must be recognized. The rabbit model could not satisfactorily simulate the non-weight-bearing activity of the human shoulder, and the experimental design was included a small number of animals and assessment indices (e.g., anatomical parameters variation measurement and long-term observation may provide more valuable results, and biomechanical tests should probably be involved). We also should recognize that neither the qPCR nor Western blot expression results were consistent with the expected levels, which weakened the power of the evidence. Nevertheless, the study provided primary evidence from animal experiments, which supported our hypothesis. In further studies, a larger sample size and superior animal model in combination with computer assistance could be employed. We believe that stronger evidence from clinical data could be obtained from trials with larger samples and longer follow-up time.

## Conclusion

Based on primary animal experimental evidence, postoperative malrotation of humerus shaft fractures could lead to consequent rotator cuff degeneration as well as shoulder osteoarthritis. However, further evidence needs to be obtained.

### Ethical review committee statement

In accordance with the ethical standards of the 1964 Declaration of Helsinki, all relevant ethical safeguards were met in relation to patient or subject protection with the authorization of the institutional ethical review committee.

## Supplementary Information

Supplementary Information.
