# Supplementary material for: Postoperative malrotation of humerus shaft fracture causes degeneration of rotator cuff and cartilage
Source: Sci Rep. 2021 Sep 20;11:18596. doi: 10.1038/s41598-021-98040-6 (PMC8452695; doi:10.1038/s41598-021-98040-6)
Supplement: Supplementary file 1 — Supplementary Information. [file 41598_2021_98040_MOESM1_ESM.pdf]

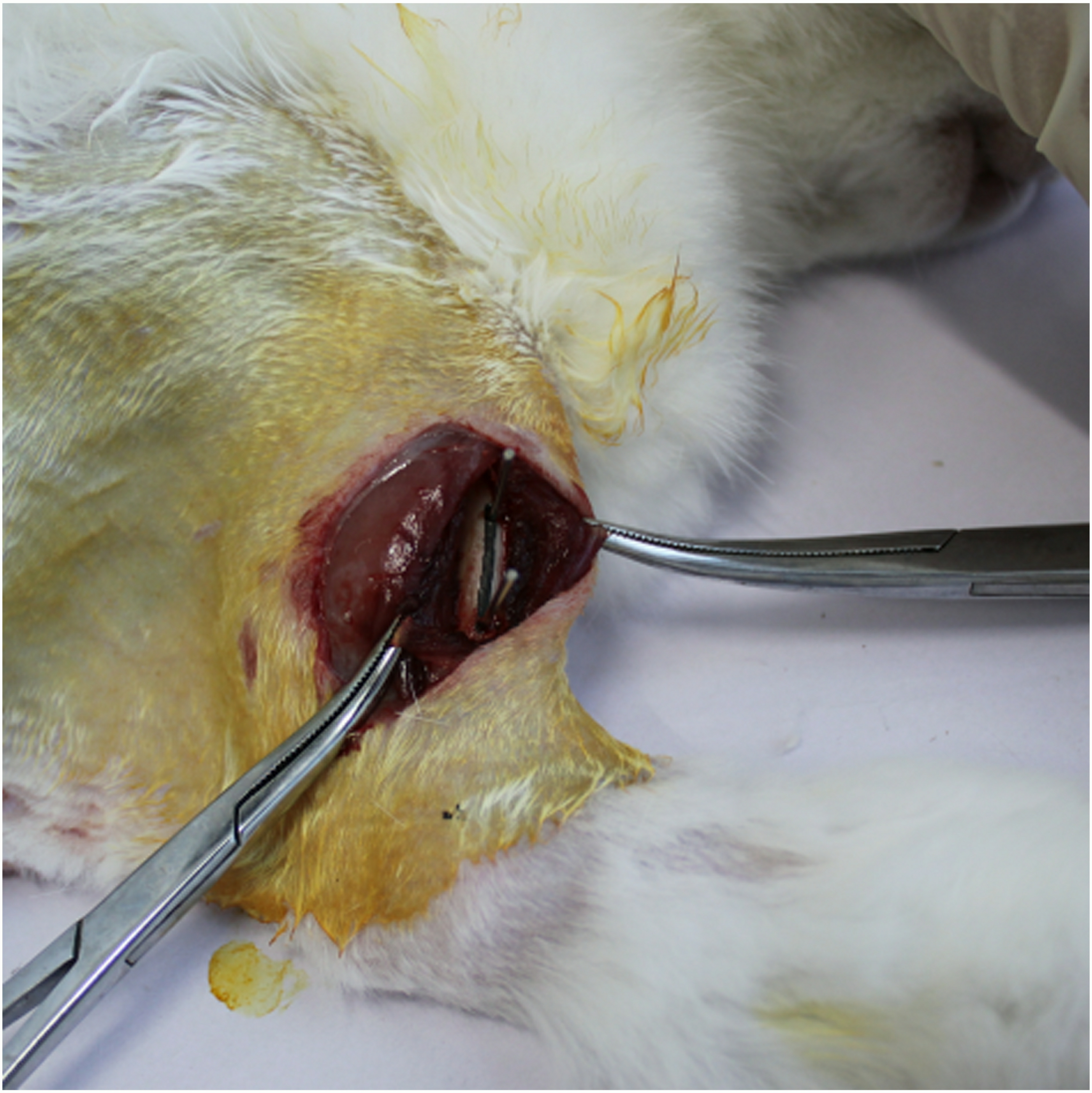

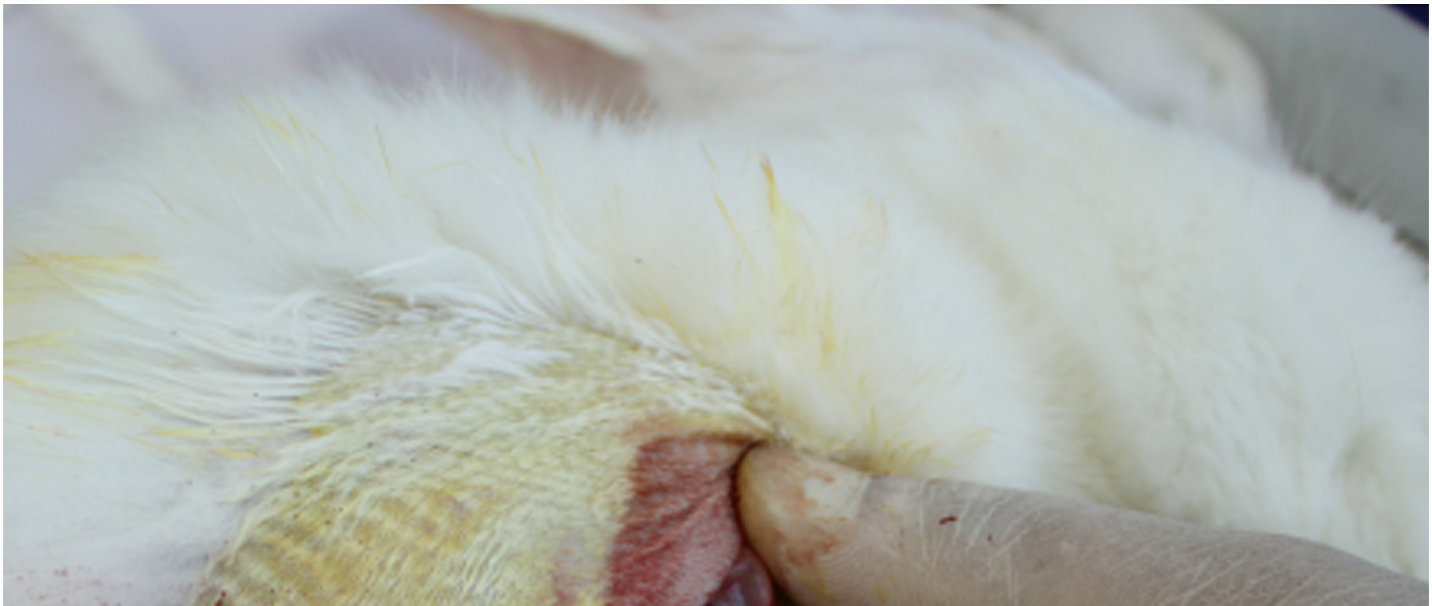

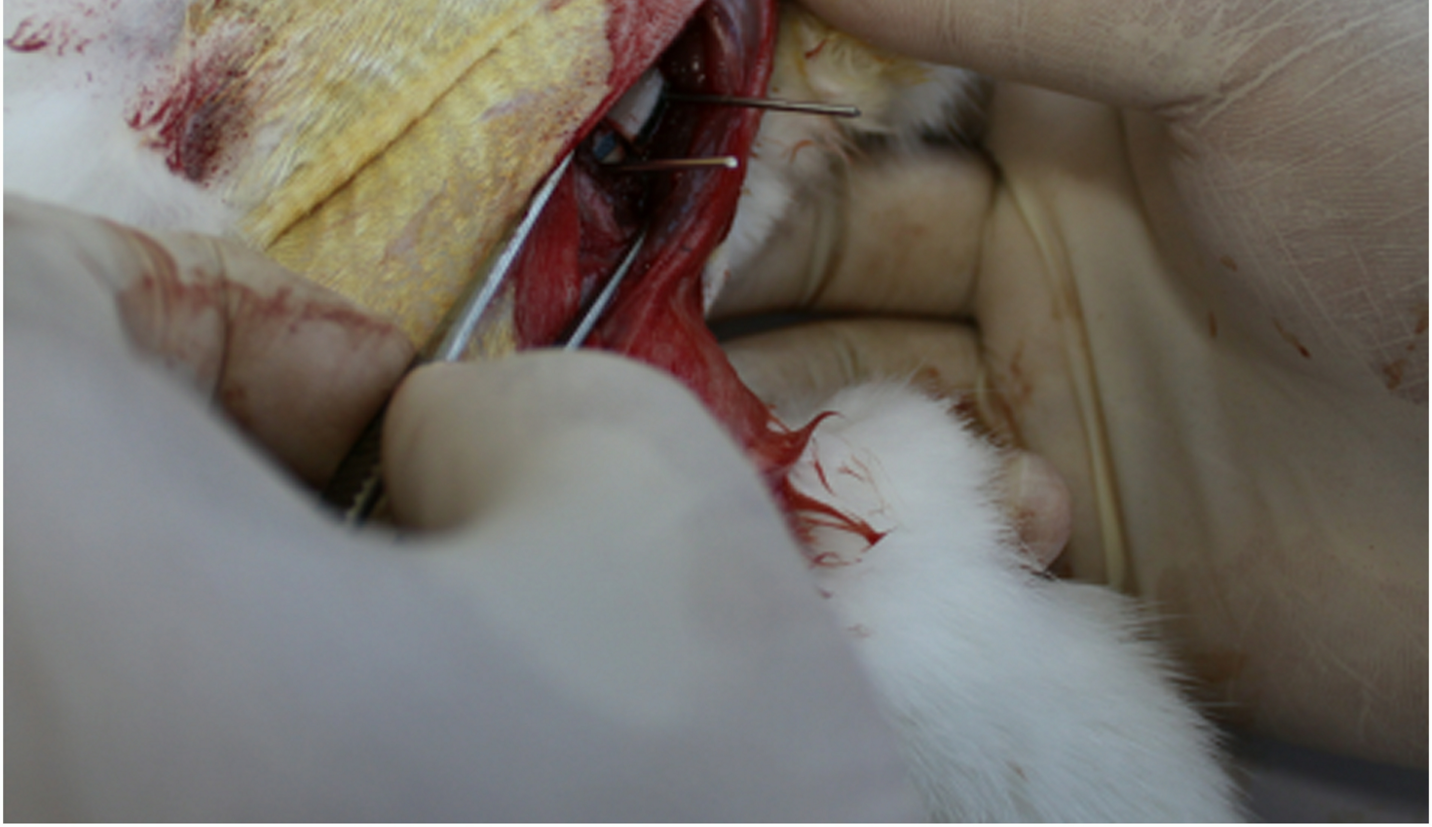

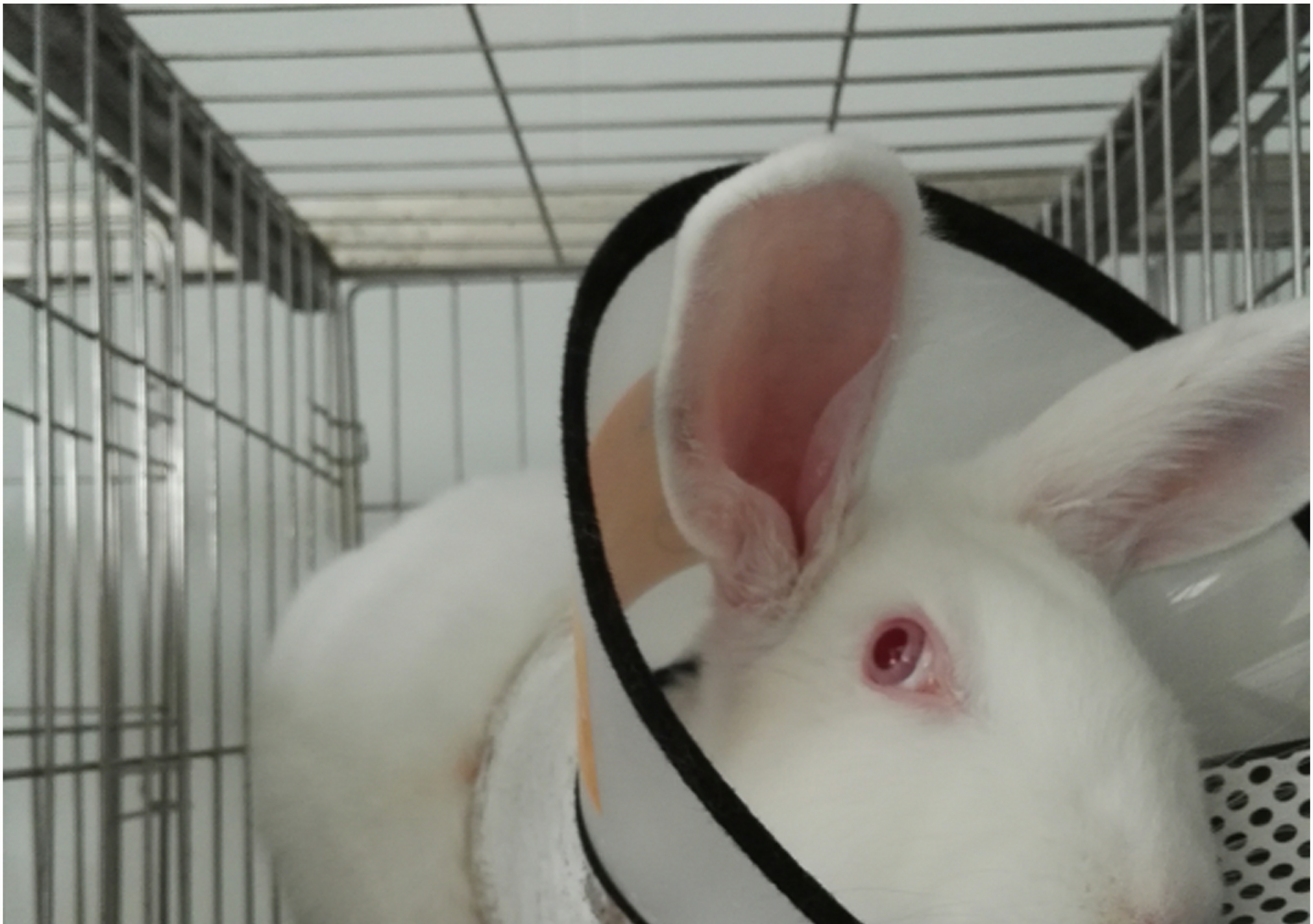

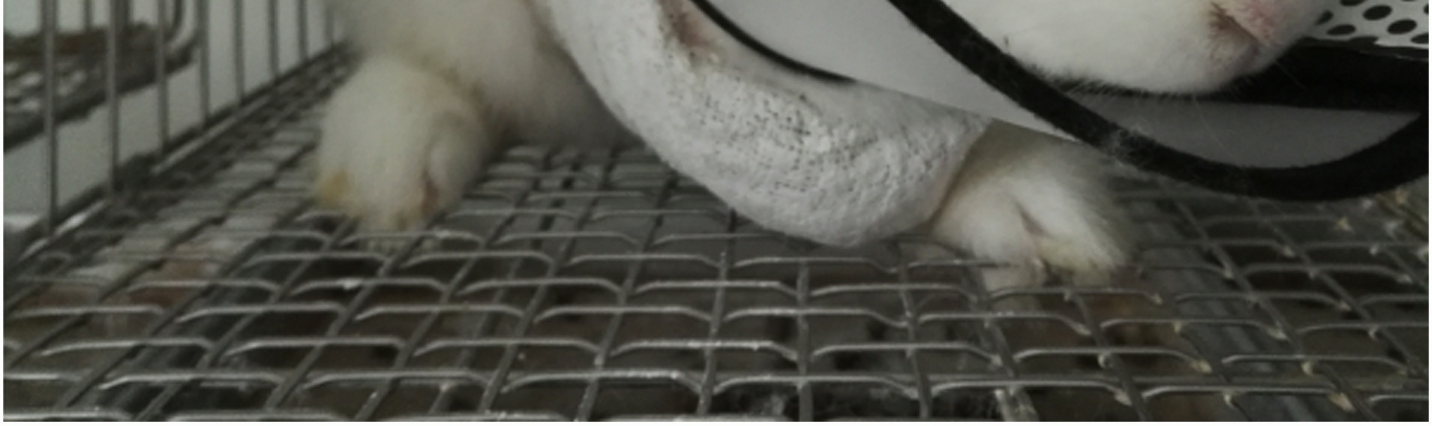

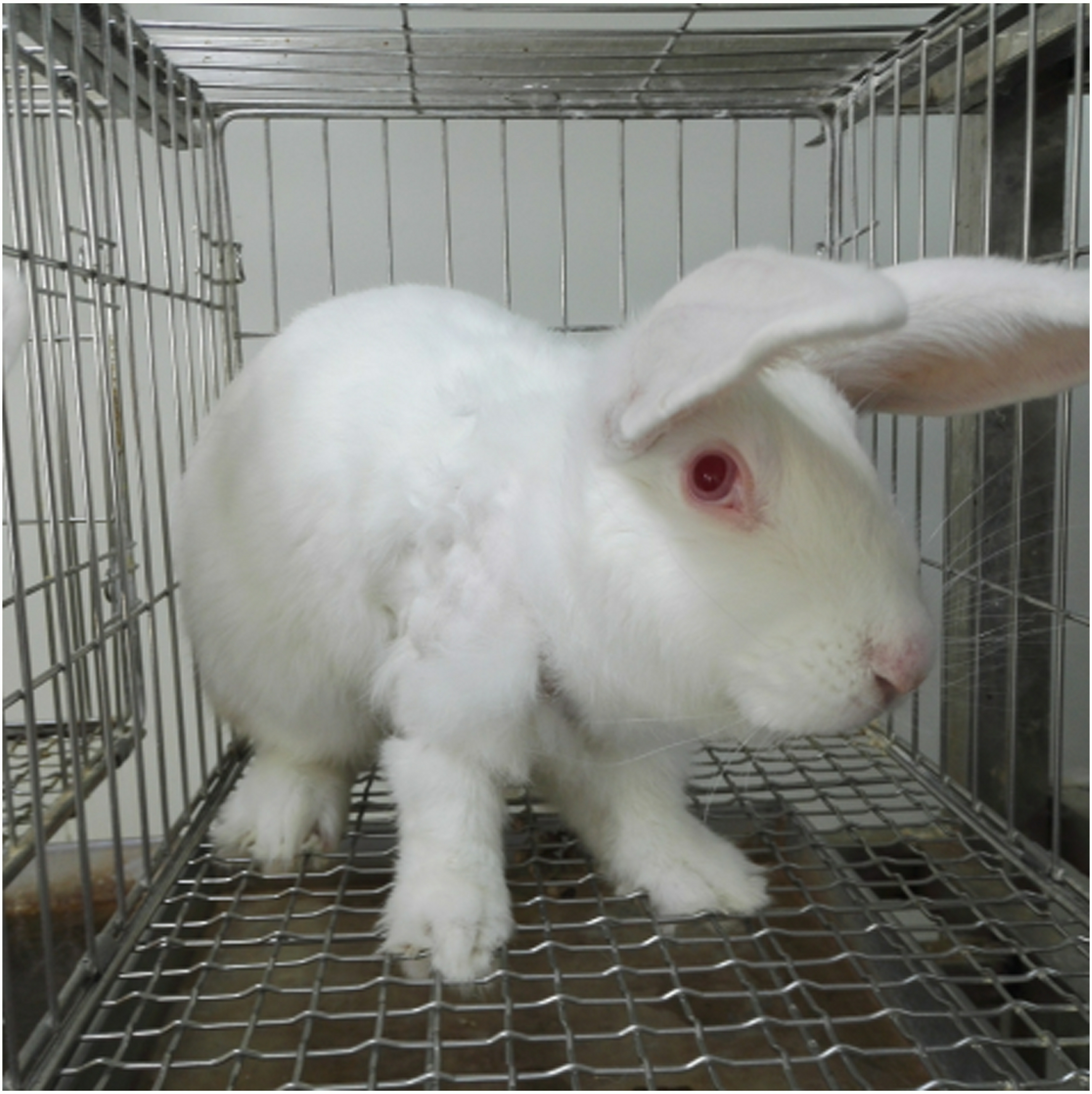

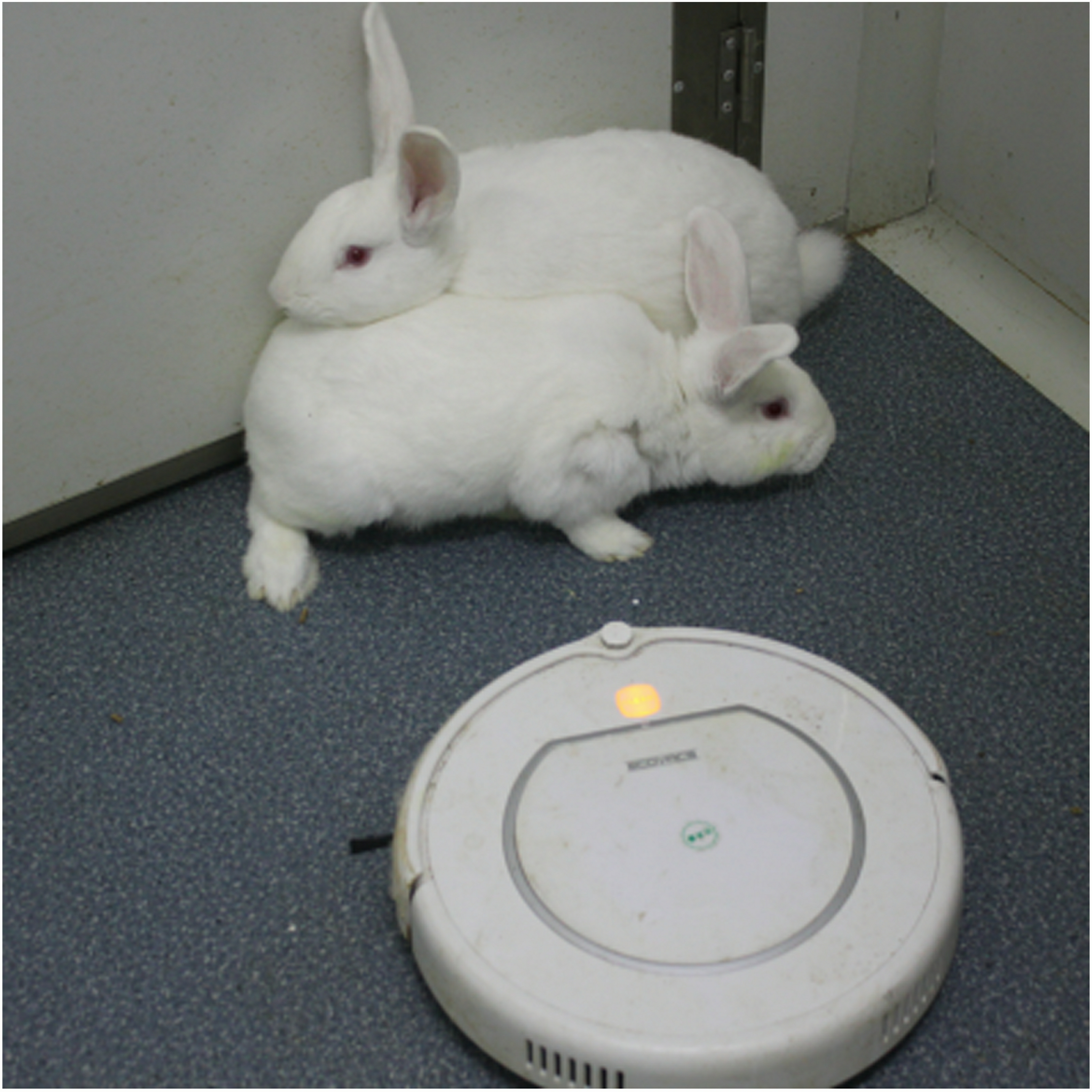

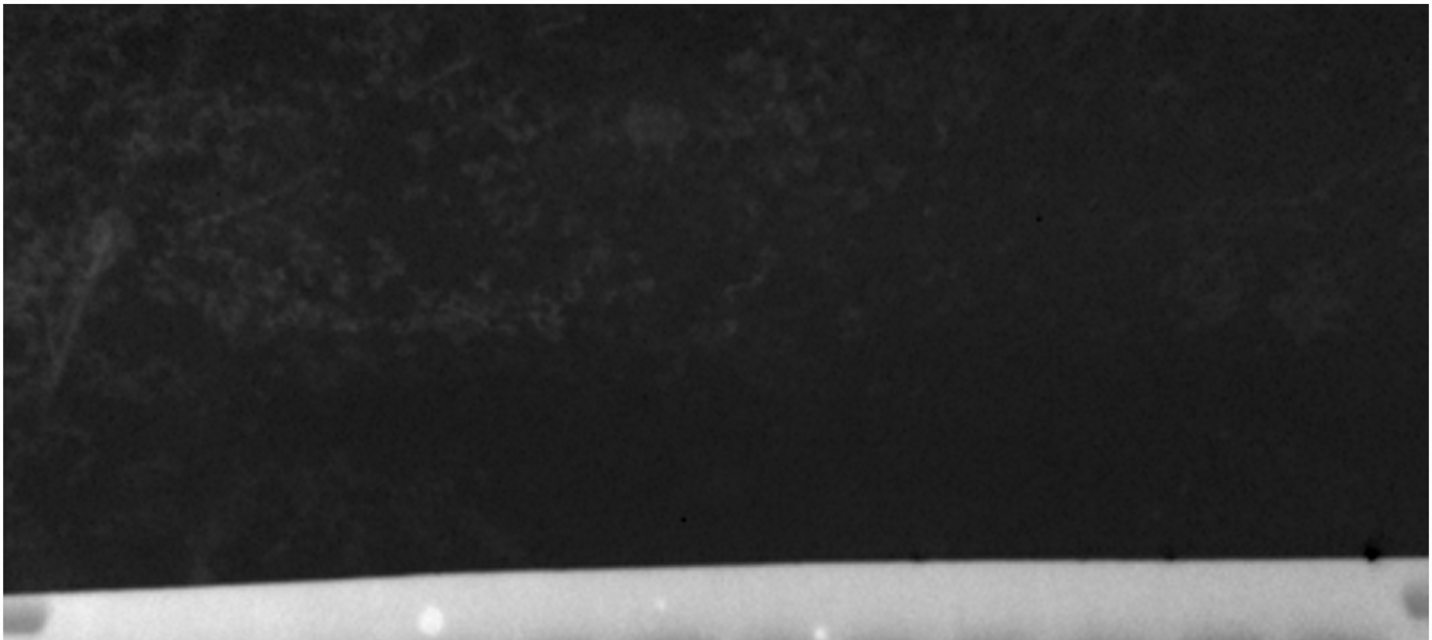

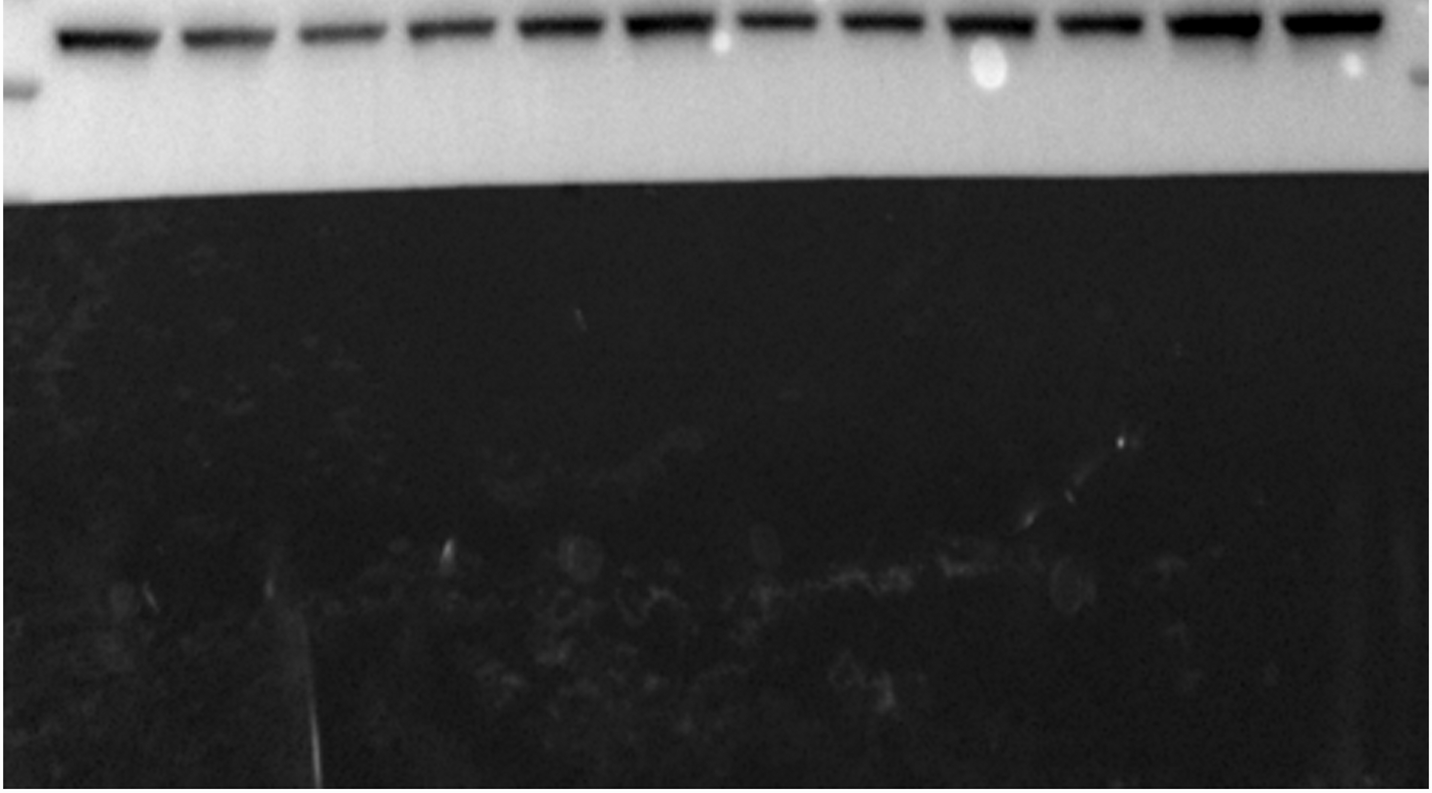

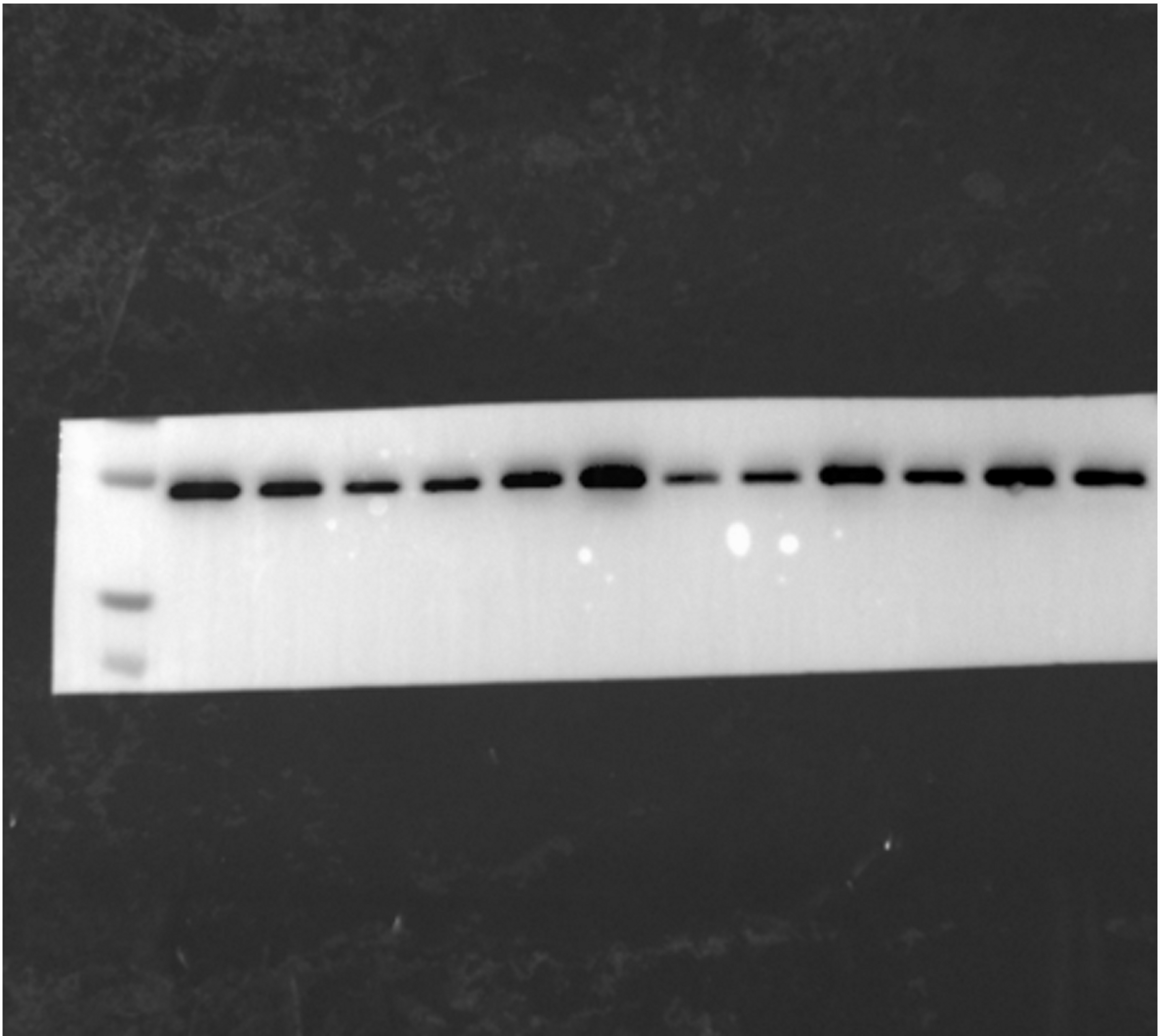

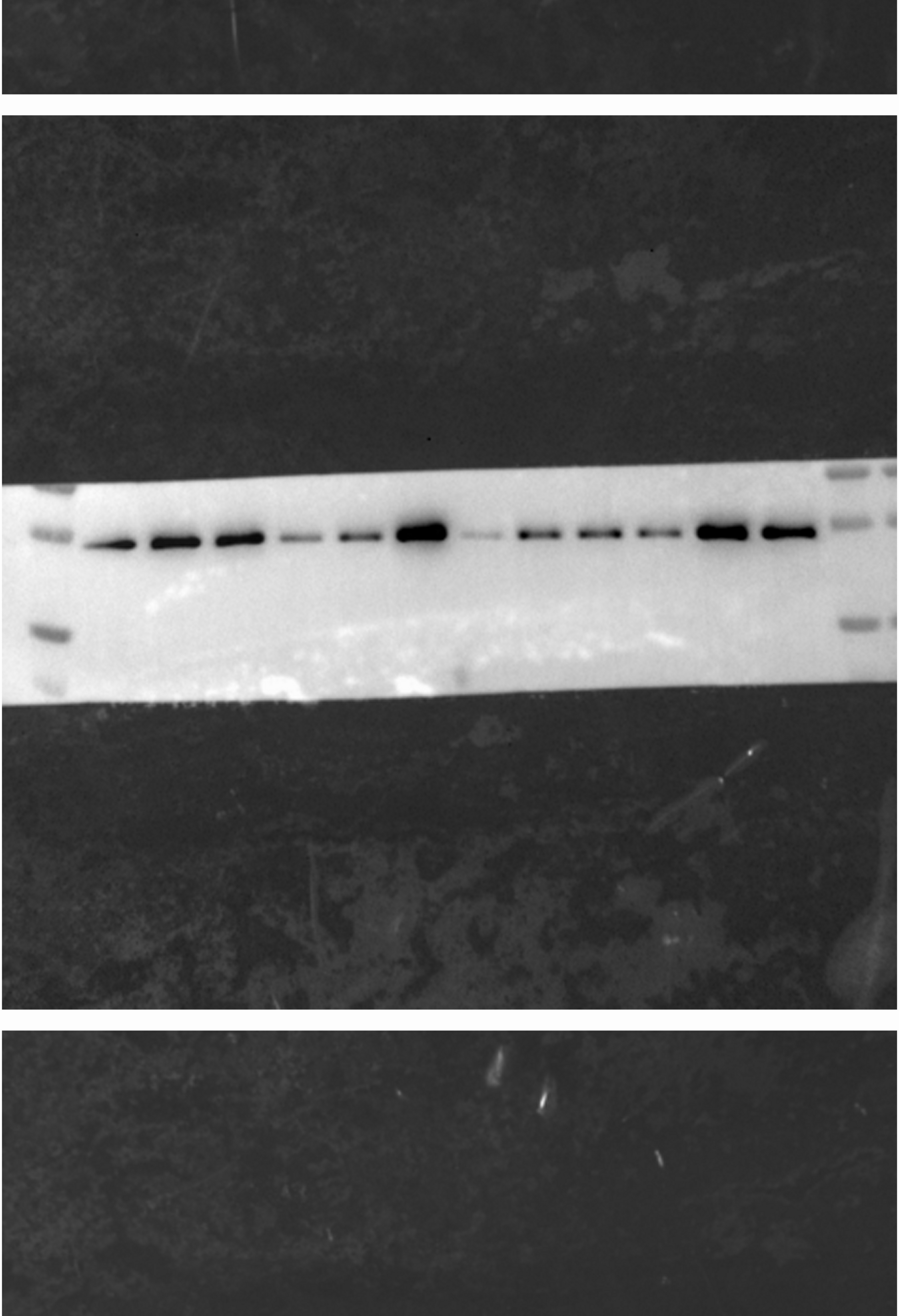

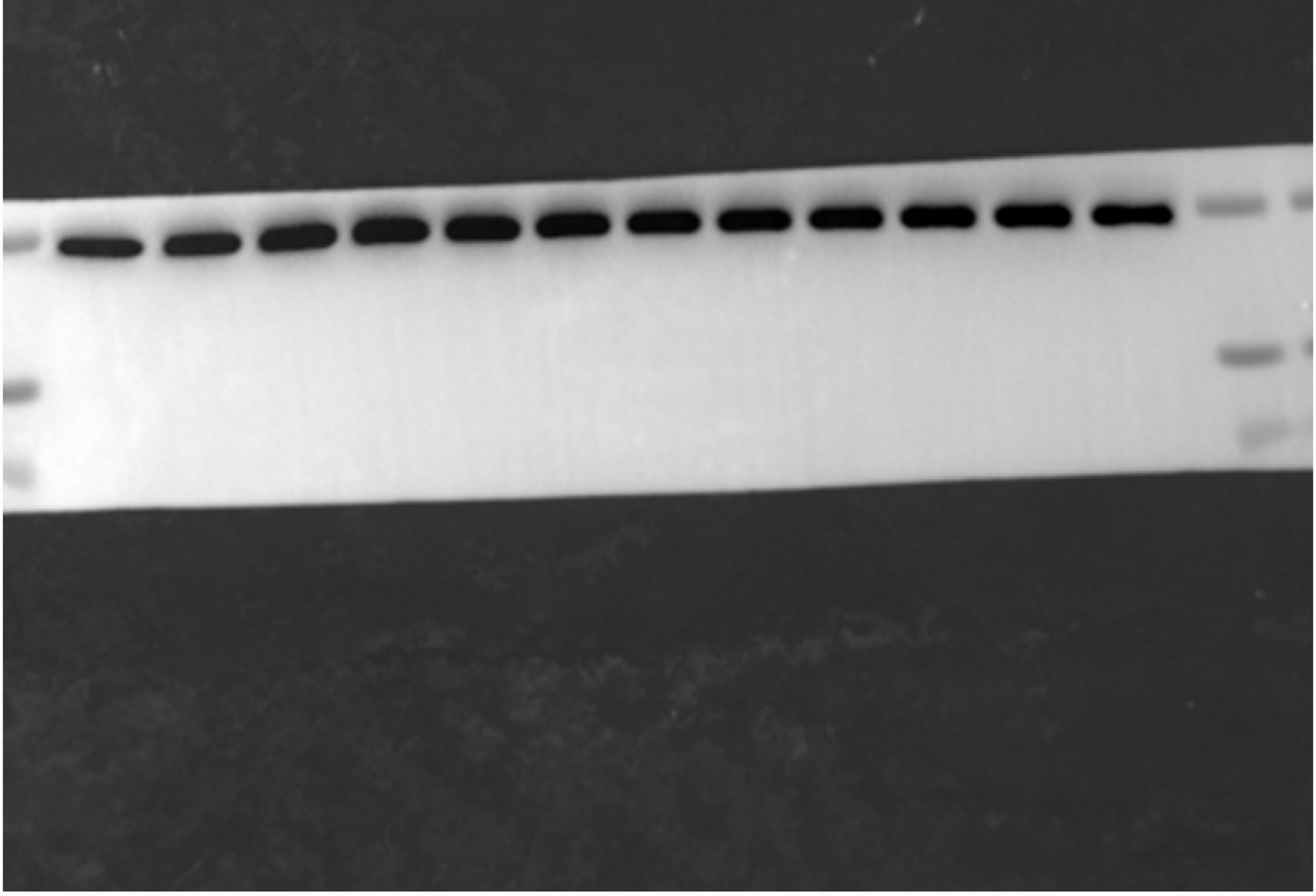

Fig1, 2: Two paralleled Kirschner wires were fixed in the distal and proximal ends of the osteotomy plane respectively, and then the proximal Kirschner wire was internally rotated along the plane perpendicular to humeral shaft axis until the target angle (20 degrees) between the two wires was attained.

Fig3: Plaster immobilization after surgery.

Fig4: Plaster removal at six weeks after surgery.

Fig5: Animals were encouraged by robots to passively move in a small room.

Fig6: Macroscopically view of Degeneration of shoulder cartilage (from group C).

Fig7: From top to bottom were the full length electrophoretic maps of MMP1, MMP13, SDF-1α and β-actin from12 samples (4 cases from each group).
